# Older people and stroke: a machine learning approach to personalize the rehabilitation of gait

**DOI:** 10.3389/fragi.2025.1562355

**Published:** 2025-05-22

**Authors:** Elvira Maranesi, Federico Barbarossa, Leonardo Biscetti, Marco Benadduci, Elisa Casoni, Ilaria Barboni, Fabrizia Lattanzio, Lorenzo Fantechi, Daniela Fornarelli, Enrico Paci, Sara Mecozzi, Manuela Sallei, Mirko Giannoni, Giuseppe Pelliccioni, Giovanni Renato Riccardi, Valentina Di Donna, Roberta Bevilacqua

**Affiliations:** ^1^ Scientific Direction, IRCCS INRCA, Ancona, Italy; ^2^ Unit of Neurology, IRCCS INRCA, Ancona, Italy; ^3^ Clinical Unit of Physical Rehabilitation, IRCCS INRCA, Ancona, Italy; ^4^ Unit of Nuclear Medicine, IRCCS INRCA, Ancona, Italy; ^5^ Unit of Radiology, IRCCS INRCA, Ancona, Italy; ^6^ Unit of Neuroradiology, IRCCS INRCA, Ancona, Italy; ^7^ Clinical Unit of Physical Rehabilitation, IRCCS INRCA, Fermo, Italy

**Keywords:** artificial intelligence, gait parameters, machine learning, medical imaging, neurology, older adults, stroke, rehabilitation

## Abstract

**Introduction:**

Stroke is a significant global public health challenge, ranking as the second leading cause of death after heart disease. One of the most debilitating consequences for stroke survivors is the restriction of mobility and walking, which greatly impacts their quality of life. The scientific literature extensively details the characteristics of post-stroke gait, which differs markedly from physiological walking in terms of speed, symmetry, balance control, and biomechanical parameters. This study aims to analyze the gait parameters of stroke survivors, considering the type of stroke and the affected cerebral regions, with the goal of identifying specific gait biomarkers to facilitate the design of personalized and effective rehabilitation programs.

**Methods:**

The research focuses on 45 post-stroke patients who experienced either hemorrhagic or ischemic strokes, categorizing them based on the location of brain damage (cortical-subcortical, corona radiata, and basal ganglia). Gait analysis was conducted using the GaitRite system, measuring 39 spatio-temporal parameters.

**Results:**

Statistical tests revealed no significant differences, but Principal Component Analysis identified a dominant structure. Machine learning (ML) algorithms—Random Forest (RF), Support Vector Machine (SVM), and k-Nearest Neighbors (KNN)—were employed for classification, with RF demonstrating superior performance in accuracy, precision, recall (all exceeding 85%), and F1 score compared to SVM and KNN. Results indicated ML models could identify stroke types based on gait variables when traditional tests could not. Notably, RF outperformed others, suggesting its efficacy in handling complex and nonlinear data relationships.

**Discussion:**

The clinical implication emphasized a connection between gait parameters and cerebral lesion location, notably linking basal ganglia lesions to prolonged double support time. This underscores the basal ganglia’s role in motor control, sensory processing, and postural control, highlighting the importance of sensory input in post-stroke rehabilitation.

## 1 Introduction

Stroke is a global public health issue, representing the second leading cause of death after heart attack (Khalid et al., 2023) and the sixth highest cause of burden of disease worldwide in terms of disability adjusted life years (Feigin et al., 2025; Johnson et al., 2016). The burden of stroke is projected to increase, with deaths expected to rise by 50% between 2020 and 2050, from 6.6 million to 9.7 million annually (Feigin et al., 2023). Restriction of mobility and walking is a major limitation that stroke survivors typically experience. About 80% of stroke patients are estimated to have ambulatory disability 3 months after the acute event (Govori et al., 2024; Teodoro et al., 2024). Recent studies (Roelofs et al., 2023; Blennerhassett et al., 2012) highlight that despite improvements in gait recovery, about 70% of community-dwelling stroke survivors experience falls within a year, often due to balance loss while walking.

Scientific literature extensively describes the features of post-stroke gait, which differs from physiological walking in terms of speed, symmetry, balance control and biomechanical aspects. Decreased walking speed is a typical sign of post-stroke gait and recent assessments confirm that gait velocity for individuals with post-stroke impairment ranges from approximately 0.18 to 1.03 m/s, whereas healthy age-matched adults average 1.4 m/s (Mohan et al., 2021; Darcy et al., 2024). This substantial difference in walking speed alone accounts for a significant proportion of the variance between post-stroke and physiological gait patterns. Current research confirms that self-selected walking speeds for stroke survivors remain below the 0.80 m/s threshold considered necessary for effective community ambulation (Middleton et al., 2015). Walking speed has been validated as a critical outcome measure for motor recovery, with improvements typically observed from 3 months up to 12–18 months post-stroke, while other functional measures may plateau earlier (Selves et al., 2020; Lee et al., 2015). Temporal and spatial inter-limb asymmetries significantly contribute to the variance in post-stroke gait compared to physiological walking (Lee et al., 2025). Recent literature confirm that spatiotemporal characteristics of post-stroke gait typically include reduced step or stride length and increased step length asymmetry between affected and unaffected sides (Patterson et al., 2010; Wonsetler and Bowden, 2017). A significant negative association is reported between the asymmetry ratios (affected side/unaffected side) of stance time, swing time and stride length with self-selected walking speed (Patterson et al., 2010; Hulleck et al., 2022), as well as an association between greater reduction in stride length and slower walking at patient’s highest-comfortable speed is also described (Beaman et al., 2010). While general gait parameters may improve over time, asymmetrical patterns often persist, presenting a challenge for rehabilitation strategies. Inter-limb spatio-temporal asymmetries of post-stroke gait also correlate with impaired standing balance control (Teodoro et al., 2024; Lewek et al., 2014), which is a further feature of gait in stroke outcomes. Traditional clinical assessments remain valuable but have limitations in capturing subtle gait abnormalities (Kokkotis et al., 2023). For this reason, instrumented gait analysis has become the gold standard for research settings, providing accurate and reliable biomechanical evaluation of key parameters including spatiotemporal, kinematic, and kinetic measures (Hulleck et al., 2022). These laboratory-based assessments typically employ motion capture systems, force platforms and sensor-embedded walkways.

A significant emerging trend is the application of artificial intelligence in predicting functional outcomes and personalizing rehabilitation programs. Machine learning techniques are being developed to identify relationships between stroke characteristics and gait parameters, supporting more tailored and effective rehabilitation strategies (Kokkotis et al., 2023; Jeon et al., 2024; Harari et al., 2020).

The main objective of this paper is to analyze the gait parameters that characterized the stroke survivors, on the basis of type of stroke and interested cerebral area, with the aim of identifying peculiar gait biomarkers for the implementation of personalized and effective rehabilitation programmes. In addition, the secondary aim is to build, tune and test specific machine learning techniques to identify an accurate stratification of the area of stroke damage based on spatio-temporal gait parameters. In this clinical scenario, Artificial Intelligence (AI) could play a crucial role for underpinning the relationship between stroke and gait parameters and thus support a better management of the post-stroke patients by predicting functional outcomes (Kokkotis et al., 2023; Jeon et al., 2024).

## 2 Materials and methods

### 2.1 Sample description

The study population consisted of patients with stroke, admitted to the Physical Rehabilitation Unit, IRCCS INRCA, in the Ancona hospitals between 2011 and 2018. Study participants met the following inclusion criteria: 1) a history of stroke (whether ischemic or hemorrhagic) in the previous one-2 weeks; 2) age ≥65 years; 3) the ability to give informant consent; 4) a stable clinical condition; 5) the ability to walk without support or at most with only one support. The study was approved by the Ethic Committee of the IRCCS INRCA and was conducted in accordance with Helsinki Declaration. All included participants provided written informed consent to participate in the study. The trial has been registered on ClinicalTrials.gov with the number NCT01397682).

### 2.2 Procedure

Each stroke patient was assessed at admission and at discharge with the following rating scales: Motricity Index (MI) for motor functions (Cameron and Bohannon, 2000), Functional Independence Measure (FIM) for activities of daily living (Ward et al., 2011), Standing (Bohannon, 1989), Trunk Control Test (Monticone et al., 2019), Functional Ambulation Category (FAC) (Mehrholz et al., 2007). In addition, instrumental gait analysis was done with the GaitRite system. GaitRite is a pressure-sensitive sensorized mat, approximately 8.2 m long, that measures the spatio-temporal parameters of walking. The evaluation with GaitRite system (Menz et al., 2004) was carried out when patients were able to walk with one support and at discharge. The subjects performed the test wearing comfortable shoes. For each subject, height, weight and leg length (from the greater trochanter to the floor, passing through the lateral malleolus) were measured. Each subject was then asked to walk at a self-selected comfortable speed. The subjects performed three trials and the parameters were averaged over the three trials performed. Each trial began and ended approximately 2 m from the walkway, so that acceleration and deceleration occurred before and after the walkway. Parameters obtained by the GaitRite are described in Supplementary Appendix A. Before admission to the Physical Rehabilitation Unit, each patient had undergone a brain computerized tomography (CT) scan to identify location, extension and nature of the stroke lesion, whether ischemic or hemorrhagic, by acquiring axial scans. CT used were GE Bright Speed 16-slice MDCT and GE Revolution GSI 64-slice MDCT. Some of the patients with negative CT at the onset of symptoms were evaluated by magnetic resonance imaging (MRI) on Philips Ingenia 1.5 T. Patients with a brain lesion highlighted on CT or MRI were divided into three groups based on the area of brain damage involved, thus classifying the lesions according to their depth into cortical-subcortical (class 1), white matter. i.e., centrum semiovale and corona radiata (class 2), and basal ganglia lesions (class 3).

### 2.3 Statistical analysis

**FIGURE 1:**
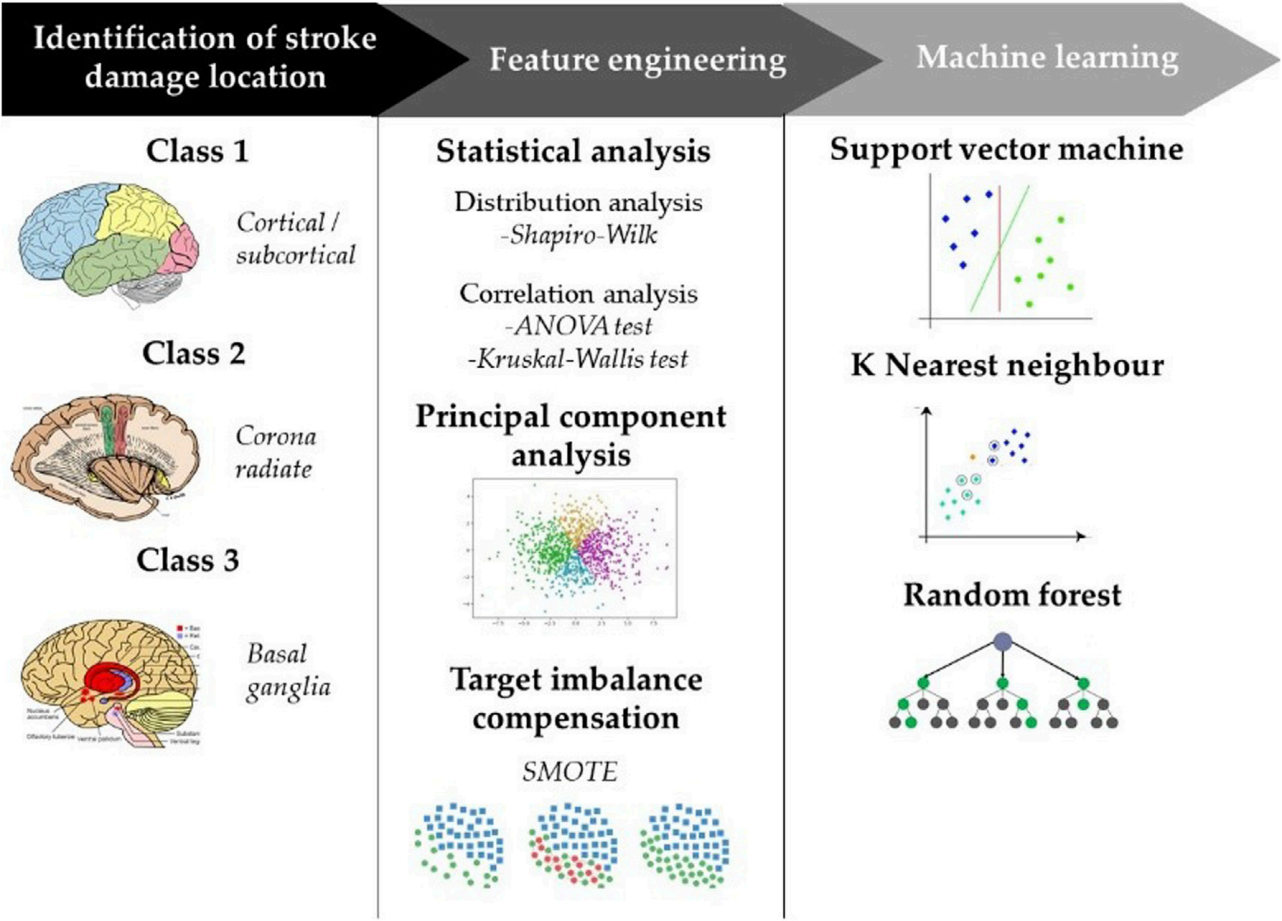
Flowchart of the adopted methodology.

Descriptive data (age, side of the lesion, Motricity Index, Holden scale Functional Independence Measure, Standing, Motricity Index for the upper and the lower limb) were presented as mean and standard deviation (SD) for continuous variables or numbers (percentage) for categorical ones. Moreover, thirty-nine gait characteristics were statistically analyzed. All the methodology steps are depicted in the flowchart in Figure 1. Since we had a small sample size, determining the distribution of the variables was important for choosing a most appropriate statistical method. In line with this, Shapiro-Wilk test was performed and, for demographic data and clinical scales, it did not show evidence of non-normality. Based on this, we decided to use a parametric test. For features whose test gives significance values greater than 0.05, a visual inspection was performed to understand the distribution of the data. The ANOVA test was used to calculate the differences between the means of each normally distributed variable while the Kruskal-Wallis test was performed on the not normally distributed variables. The result of the tests was compared with an analysis of correlation coefficients calculated pairwise among the features. This provides insight into the redundancy of the information content contributed by the totality of features and an understanding of which of the features can be neglected as the analysis continues.

### 2.4 Data pre-processing and feature engineering

Data are standardized by transforming numerical variables to have zero mean and unit variance.

Principal component analysis (PCA) was used to select the most significant features among the set of parameters that characterize the gait task. The contribution of each feature to these principal components is expressed by the magnitude of its corresponding coefficient. For each PC, the features that were given a coefficient >0.3 were reported. The results of PCA were compared with those obtained from pairwise correlation analysis so as to ensure the correct feature selection and consequently, the right number of components to be used for training machine learning algorithms.

Further, PCA was exploited to reduce dimensionality by selecting a subset of principal components that explain a significant portion of the data variance. By retaining the most informative components exclusively, we can reduce the dimensionality of the dataset while minimizing the loss of information.

### 2.5 Data augmentation

The Synthetic Minority Over-Sampling TEchnique (SMOTE) (Chawla et al., 2002) was employed as a data augmentation strategy to tackle the issue of imbalanced target variables. The use of SMOTE as a data augmentation technique provides a robust solution to the problem of imbalanced target variables, contributing to the generation of more reliable and accurate predictive models. Majority class–class 1- is composed of 27 subjects, while the minority classes consist of 7 (class 2) and 12 (class 3) samples. The generation of synthetic samples, conducted with meticulous care to uphold the fundamental patterns and distinguishing attributes inherent in the minority class, consisted in generating 19 new samples to balance class 2 and 15 samples for class 3. After augmentation, the dataset thus went from an instance size of 46 to 81. To ensure the quality of the oversampling process, we compared the statistical properties of the original dataset with those of the synthetic samples generated by SMOTE. In particular, we analyzed the feature distributions, the consistency of principal components and feature correlation. In detail, the mean and standard deviation of key gait parameters were compared before and after SMOTE application to confirm that the synthetic data did not introduce significant bias. Principal components were compared before and after SMOTE checking if the synthetic data followed the distribution of the original data without introducing artificial distortions. Finally, the correlation structure between gait parameters was preserved after oversampling, ensuring that the relationships between features remained consistent.

### 2.6 Machine learning

New PC features are used as input for machine learning algorithms in order to predict over three classes. In this study, Random Forest (RF), Support Vector Machine (SVM), and k-Nearest Neighbors (kNN) algorithms were adopted. In the RF algorithm, it was chosen 5 as minimum number of samples required in a tree node for considering a split and further dividing the node into smaller subsets and 250 as optimal number of tree population, above which no improvement in classification performance is noticeable. No restriction is imposed about the maximum number of hierarchical splits in the tree structure. SVM was chosen because of its versatility and efficient handling of moderate-sized datasets and its ability to control model complexity. In this regard, the hyperparameters that were chosen as optimal by grid search optimization are C = 2.5 and γ = 0.8. kNN architecture is built with a specific focus on parameter selection. The key parameters chosen are the number of neighbors (5) and the number of subspaces (3). For each subspace, specific parameters are defined. In order to evaluate the performance of classification models, we employed a 10-fold cross-validation procedure. In this way, the dataset is divided into distinct segments or folds, where one-fold is set aside as the test dataset and the remaining folds are used for training the classification model. This approach allows us to gain reliable insights into the model’s performance and its ability to handle new and unseen data, enhancing the overall validity of our findings. The performance of each model is then calculated for each fold and averaged to provide a comprehensive assessment of its effectiveness This process is repeated multiple times, with each fold serving as the test dataset exactly once. The performance of each model is then calculated for each fold and averaged to provide a comprehensive assessment of its effectiveness. Four metrics were calculated to interpret classification performance. Formulas of Accuracy (Equation 1), Recall (Equation 2), Precision (Equation 3) and F1 score (Equation 4) are reported below, where TP, TN, FP and FN are true positive, true negative, false positive and false negative respectively.

$$Accuracy=\frac{TP+TN}{TP+TN+FP+FN}$$ (1)

$$Recall=\frac{TP}{TP+FN}$$ (2)

$$Precision=\frac{TP}{TP+FN}$$ (3)

$$F1\;score=\frac{2*\;precision*\;recall}{precision+recall}$$ (4)

## 3 Results

For this study, 45 subjects with a hemorrhagic and ischemic stroke were considered. Clinical data were reported in Table 1. Data are reported according to stroke damage area classes.

**TABLE 1:** Sample description and clinical profile.

	Total (n = 45)	Class 1 (n = 27)	Class 2 (n = 8)	Class 3 (n = 10)	p
Gender					0.395
Female (n)	25	16	5	4	
Male (n)	20	12	3	6	
Age (mean ± sd)	82.2 ± 7.4	83.7 ± 8.1	81.0 ± 5.5	80.2 ± 6.6	0.460
MI (T0/T1)					
Upper Limb	79 ± 18.6/87.1 ± 14.2	77.4 ± 18.5/86.0 ± 11.2	72.3 ± 21.1/80.1 ± 21.9	89.1 ± 13.6/95.8 ± 9.3	0.146
Lower Limb	74.1 ± 19.7/86.5 ± 14.6	74.5 ± 21.6/86.4 ± 13.9	67.1 ± 17.0/84.5 ± 13.3	78.5 ± 16.2/88.4 ± 18.5	0.487
Holden (T0/T1)	1.6 ± 1.3/3.4 ± 1.1	1.7 ± 1.3/3.6 ± 1.1	1.6 ± 1.6/3.5 ± 0.9	1.7 ± 0.9/3.7 ± 1.1	0.993
Holden 0	12/0	8/0	3/0	1/0	
Holden 1	11/1	7/1	1/0	3/0	
Holden 2	10/13	4/9	2/2	4/2	
Holden 3	9/7	7/4	0/1	2/2	
Holden 4	4/16	2/10	2/3	0/3	
Holden 5	0/9	0/4	0/2	0/3	
FIM (T0/T1)	63.9 ± 19.2/91.7 ± 18.8	63.4 ± 18.2/89.0 ± 19.2	61.3 ± 25.2/94.6 ± 16.8	67.5 ± 18.2/96.5 ± 19.8	0.748
Standing (T0/T1)	2.3 ± 1.4/3.7 ± 0.6	2.3 ± 1.5/3.7 ± 0.5	2.1 ± 1.4/3.7 ± 0.7	2.4 ± 1.2/3.6 ± 0.9	0.921
Standing 0	9/0	8/1	1/0	0/0	
Standing 1	7/0	2/0	2/0	3/0	
Standing 2	11/6	6/3	2/1	3/2	
Standing 3	4/5	2/4	1/0	1/1	
Standing 4	14/33	9/19	2/7	3/7	

Class 1 = lesions into cortical-subcortical; Class 2 = lesions into white matter; Class 3 = lesions into basal ganglia lesions; MI, motricity index; Holden 0 = Non-ambulatory; Holden 1 = Total assistance; Holden 2 = Partial assistance; Holden 3 = Minimal assistance; Holden 4 = Independently with device; Holden 5 = Independently without device; Standing 0 = Unable to maintain upright position; Standing 1 = Maintains upright position, with widened base, but less than 30 s; Standing 2 = Maintains upright position, with widened base, more than 30 s; Standing 3 = Maintains upright position, narrow base, but less than 30 s; Standing 4 = Maintains upright position, narrow base, more than 30 s.

Statistical analysis shows no statistically significant differences in the clinical scales among three groups considered. One-way ANOVA test and non-parametric Kruskal-Wallis test returns p-values>0.05 for all variables (except for Heeloffontimer and Normvelocity) as reported in Tables 2, 3. No statistically significant differences were found among normally and not-normally distributed features.

**TABLE 2:** ANOVA test. F value and p-value are reported for each normally distributed variable.

Feature	F	p-value	Feature	F	p-value
Distance	0.981	0.382	Single support time R	0.042	0.959
Ambulation time	1.123	0.334	% double support L	1.288	0.285
Velocity	2.344	0.107	% double support R	0.608	0.548
Step count	1.744	0.186	Double support time L	0.416	0.548
Step time L	0.044	0.957	Double support time R	0.254	0.662
Step time R	1.126	0.333	Hell off time L	1.042	0.777
Cycle time L	0.334	0.718	Double support load time L	0.313	0.733
Cycle time R	0.264	0.769	Double support load time R	0.767	0.470
Support base L	1.552	0.223	Double support unload time L	0.386	0.682
Support base R	1.491	0.236	Double support unload time R	0.053	0.948
Swing time L	0.042	0.959	Stride velocity L	2.493	0.094
Stance time L	0.752	0.477	Stride velocity R	2.471	0.095
Stance time R	0.117	0.890	FAP	1.517	0.230

R = right; L = left; FAP, functional ambulation profile.

**TABLE 3:** Kruskal-Wallis test. H value and p-value are reported for each not-normally distributed variable.

Feature	H	p-value	Feature	H	p-value
Cadence	4.075	0.130	Swing time R	2.985	0.225
Step length L	4.560	0.334	Single support time L	3.730	0.155
Step length R	1.673	0.107	Toe in-out L	0.882	0.643
Step extremity L	4.635	0.186	Toe in-out R	0.161	0.923
Step extremity R	1.688	0.957	Hell off time R	7.432	0.024
Stride length L	3.620	0.333	Velocity normalized	6.723	0.035*
Stride length R	4.003	0.718			

R = right; L = left; FAP, functional ambulation profile; *p < 0.05.

This confirms that classical statistical methods are not suitable enough to identify a priori complex data patterns, hence it is necessary to explore the data with more specific methods in order to identify underlying patterns.

Applying PCA to the standardized data, it can be seen that six principal components (PCs) were sufficient to explain more than 90% of the variance of the data. Table 4 shows the combination of features that had the greatest influence on each PC. The features that have the most influence in determining the PC1 are time for the double support at right and left site while swing time for the left site and time of single support for the right site have the most effort in calculating the PC2. The remaining PCs have a smaller percentage of explained variance, meaning that their contribution in representing the original feature space is significantly less than the first two. Consequently, the features that characterize these PCs will also have a different weight in the information effort, contributing in a minor way to representing the original feature space.

**TABLE 4:** Principal components.

	PC1	PC2	PC3	PC4	PC5	PC6
Explained variance	54.22%	18.62%	8.52%	5.22%	3.24%	2.49%
Original features indexes	Double support time L/R	Swing time L, Single support time R	Step time R, Swing time R, Single support time L	Double support unload time R	Toe in-out L/R	Step extremity R, Hell off time L

R = right; L = left.

The metrics obtained from cross-validation describing the predictive performance on the three classes of all 3 ML models are shown in Table 5.

**TABLE 5:** Performance metrics in cross-validation for all 3 ML models.

	Accuracy (%)	Precision (%)	Recall (%)	F1 score (%)
RF	85.56	89.44	85.56	84.49
SVM	80.56	80.58	80.56	78.33
KNN	78.16	73.85	78.06	78.80

RF , random forest; SVM, support vector machine; kNN = k-Nearest Neighbors.

Optimized RF model demonstrated superior performance compared with optimized SVM in multiclass classification. Using a dataset of 80 instances and a three-class target, RF achieved greater than 85% accuracy, precision, recall. The F1 score obtained by the model was similarly high. On the other hand, SVM showed slightly lower performance than RF, with a slightly lower F1 score (78.33%). Finally, the KNN model showed slightly lower performance than SVM and RF, with accuracy, recall and F1 score just below 80%. RF shows best discriminating performance in classifying three classes in terms of accuracy, precision, recall and F1 score with respect to SVM and KNN.

## 4 Discussion

This paper focuses on analyzing gait parameters in stroke survivors, considering stroke type and affected cerebral area. The goal is to identify specific gait biomarkers for personalized rehabilitation.

From a clinical point of view, the present study suggests that there is a possible connection between some gait parameters and location of cerebral lesions in patients with stroke. Specifically, PC1, accounting for about 54% of total variance, resulted to associated with double support L/R. Looking at the median values of this parameter in the three groups, we can observe that it is highest in the group 3, composed of patients suffered from lesions at basal ganglia level. Therefore, even if classical statistical approaches did not find any significant difference among the three groups making up our cohort with respect to double support L/R, we can hypothesize that patients with stroke lesions involving basal ganglia tend to keep double support L/R for more time than subjects affected by ischemic or haemorrhagic infarcts in other brain regions. In general, the link between basal ganglia lesions and gait impairment was reported by several studies. For instance, a recent investigation found an association of basal ganglia micro-bleeds with both stride length and step length in patients with cerebral small vessel disease (Mao et al., 2023) It is important to underline that basal ganglia are significantly involved in motor programming (Takakusaki, 2017): this can justify a relevant difficult in the activation of gait with a consequent generally higher time of double support L/R.

During walking, the foot plays two distinct functional roles: in the double support phase, it primarily acts as a “sensory organ,” acquiring postural inputs to prepare for the subsequent swing phase. Our study identified bilateral double support time (R/L) as the most influential parameter in the variance of gait patterns, with Principal Component 1 (PC1) explaining 54.22% of the total variance. Specifically, our results showed that patients with basal ganglia lesions exhibited the highest median double support time, suggesting a compensatory mechanism for impaired postural control (Mao et al., 2023; Takakusaki, 2017; Perry, 1992).

This finding aligns with previous research highlighting the role of the basal ganglia in motor programming and postural stability. While these structures do not have direct sensory projections, they process sensory information indirectly, integrating it into motor output. The prolonged double support time observed in our study suggests that patients with basal ganglia damage may rely more on increased stance duration to stabilize their gait, compensating for deficits in dynamic balance. This is consistent with existing evidence linking basal ganglia microbleeds to altered stride and step length in cerebrovascular disease patients (Takakusaki, 2017; Bhatia and Marsden, 1994; Brown et al., 1997; Hemami and Moussavi, 2014; Wilson et al., 2019). Furthermore, our machine learning classification results reinforce this association: the Random Forest model achieved over 85% accuracy, precision, and recall in distinguishing between stroke lesion locations based on gait parameters. Given that double support time was among the primary discriminative features, our findings suggest that this metric serves as a key biomarker for identifying stroke-related gait impairments. From a clinical perspective, these insights highlight the potential role of sensory-based rehabilitation strategies for stroke survivors with basal ganglia damage. Previous studies have demonstrated that sensory input training significantly improves motor function and balance control in post-stroke patients (Takakusaki, 2017; Jo et al., 2023). Our results suggest that rehabilitation programs should incorporate proprioceptive and postural control exercises, targeting the specific gait compensations observed in patients with basal ganglia lesions.

Therefore, intercepting the brain areas mostly involved in both motor control and sensory processing, including primarily basal ganglia, by means of the artificial intelligence algorithm used in the present study, might guide rehabilitation treatment to tailor-made programs, according to principles of precision medicine. This could be useful in the setting of post-stroke rehabilitation in the view of both predictions of recovery and programming interventional treatment strategies, which could be differentiated depending on the location of stroke lesions.

Our study differed in methodology and result with respect to current existing scientific research in this field. While different studies, reported in the review by Kokkotis et al. (2023), (Jo et al., 2023), use clinical and imaging data to predict medium-to long-term functional outcomes, our work focuses on the use of spatiotemporal gait parameters to identify biomarkers related to brain injury, with the goal of personalizing rehabilitation, especially explaining possible hidden relationships between gait parameters and cerebral area involved in stroke. In addition, although the review reports extensive use of deep learning models, our study highlights the robustness of more interpretable models such as Random Forest and SVM, supported by PCA analysis for the selection of the most relevant features. This approach provides a solid basis for the development of rehabilitation strategies based on gait parameters, an aspect less explored in the studies cited in the review.

However, the present study has some limitations. First, the cohort size (45 patients) is relatively small and the patients are not equally distributed among the three classes considered in the study, i.e., class 1 cortical-subcortical lesions, class 2 white matter lesions and class 3 basal ganglia lesions. However, the use of data augmentation strategy is a validated method to reduce risks of bias linked to class imbalance. The relatively small number of patients with basal ganglia lesions in the original dataset (n = 12) represent a point of weakness. While SMOTE was applied to balance the class distribution, we acknowledge that the inclusion of synthetic samples (n = 15) may have influenced the observed association between double support time and basal ganglia damage. Future studies with larger and more balanced datasets are needed to confirm these findings without the need for oversampling techniques. A second limitation is linked to a not precise sub-classification of cortical lesions. To this regard, it is important to underline that different cortical areas play different roles in motor control (Chen et al., 2018). Therefore, a classification of cortical lesions could be of great interest in order to measure the specific impact of each cortical area in motor impairment. Unfortunately, in our cohort, due to the relatively small sample size, it was not possible to sub-classify cortical lesions in the view of using a machine learning approach, because each subgroup would have been too small to test AI algorithms.

In summary, our study suggests that, using a ML approach, it is possible to correctly recognize the location of a stroke lesion based on Gait Rite parameters. Further larger prospective studies incorporating GaitRite data at the end of the rehabilitation program will be needed to confirm these results and to verify their potential impact on routine clinical practice.

## 5 Conclusion

Our study demonstrates that principal component analysis (PCA) can effectively reduce the dimensionality of gait data while preserving key variance, which can then be used as input features for machine learning models. The Random Forest (RF) algorithm showed superior performance in multiclass classification of stroke patients based on gait parameters, with accuracy, precision, recall, and F1 scores all exceeding 85%. This suggests that RF is highly effective for this type of biomedical informatics application.

For developers and users of biomedical informatics methods, incorporating dimensionality reduction techniques like PCA can enhance the performance of machine learning models by focusing on the most informative features. Selecting robust algorithms is crucial, and in our study, the Random Forest algorithm outperformed Support Vector Machine (SVM) and k-Nearest Neighbors (kNN), indicating its robustness in handling complex biomedical data. It should be considered a strong candidate for similar classification tasks.

Understanding the clinical implications of data features, such as the association of basal ganglia lesions with prolonged double support time, can provide valuable insights that enhance model interpretability and clinical relevance. Ensuring comprehensive and high-quality data collection, including detailed clinical profiles and precise gait analysis, is critical for developing accurate predictive models.

By integrating these recommendations, developers and users can improve the effectiveness and applicability of biomedical informatics methods in clinical settings, particularly for personalized rehabilitation strategies for stroke survivors.

## Data Availability

The raw data supporting the conclusions of this article will be made available by the authors, without undue reservation.
