# Supplementary material for: Older people and stroke: a machine learning approach to personalize the rehabilitation of gait
Source: Front Aging. 2025 May 22;6:1562355. doi: 10.3389/fragi.2025.1562355 (PMC12142055; doi:10.3389/fragi.2025.1562355)
Supplement: Supplementary file 1 [file Table1.docx]

| **Feature** |  |
| --- | --- |
| Distance | Distance from the heel center of the first footprint to the heel center of the last footprint [cm]. |
| Ambulation time | It is the time elapsed between first contact of the first and the last footfalls [sec]. |
| Velocity | It is the ratio of Distance and Ambulation time. [cm/sec] |
| Velocity normalized | It is obtained after dividing the Velocity by the Average Leg Length [(left leg length + right leg length)/2]. |
| Step count | Number of steps between first contact of the first and the last footfalls. |
| Cadence | The number of steps taken in one second. |
| Step length | It is measured along the line of progression, from the heel center of the current footprint to the heel center of the previous footprint on the opposite foot [cm]. |
| Stride length | It is measured on the line of progression between the heel points of two consecutive footprints of the same  foot (left to left, right to right) [cm]. |
| Step time | It is the time elapsed from first contact of one foot to first contact of the opposite foot [sec]. |
| Step extremity | It is defined as the Step Length divided by the Leg Length of the same leg. |
| Cycle time | It is the elapsed time between the first contacts of two consecutive footfalls of the same foot. |
| Support base | It is the vertical distance from heel center of one footprint to the line of progression formed by two footprints of the opposite foot. |
| Swing time | It is initiated with toe off and ends with heel strike. It is the time elapsed between the Last Contact of the current footfall to the First Contact of the next footfall on the same foot [sec]. |
| Stance time | It is initiated by heel contact and ends with toe off of the same foot. It is the time elapsed between the First Contact and the Last Contact of two consecutive footfalls on the same foot. |
| Single support time | It is the time elapsed between the Last Contact of the current footfall to the First Contact of the next footfall of the same foot [sec] |
| % double support | Total double support is the sum of the initial double support and the terminal double support [% of gait cycle time]. Initial double support occurs from heel contact of one footfall to toe-off of the opposite footfall. Terminal double support occurs from opposite footfall heel strike to support footfall toe-off. |
| Double support time | Total double support is the sum of the initial double support and the terminal double support sec]. Initial double support occurs from heel contact of one footfall to toe-off of the opposite footfall. Terminal double support occurs from opposite footfall heel strike to support footfall toe-off. |
| Heel off time | It is the time that the last sensor goes off in any quadrilateral. |
| Double support load time | Time of double support loading. |
| Double support unload time | Time of double support unloading. |
| Stride velocity | It is obtained after dividing the Stride Length by the Stride Time. |
| Toe in-out | It is the angle between the line of progression and the midline of the footprint. Angle is zero if the geometric mid-line of the footprint is parallel to the line of progression (that is the line connecting the heel centers of two consecutive footfalls of the same foot); positive, toe-out, when the mid-line of the footprint is outside the line of progression and negative, toe-in, when inside the line of progression. |
| FAP | Functional Ambulation Performance Score |
